# Invasive thyroglossal duct cyst papillary carcinoma: a case report

**DOI:** 10.1186/1752-1947-3-9308

**Published:** 2009-12-01

**Authors:** Leila Aghaghazvini, Habib Mazaher, Hashem Sharifian, Shirin Aghaghazvini, Majid Assadi

**Affiliations:** 1 Department of Radiology, Amiralam Hospital, Tehran University of Medical Science, Tehran, Iran; 2 Department of Nuclear Medicine and Oncology, The Persian Gulf Biomedical Research Institute, Busheher University of Medical Sciences, Bushehr, Iran

## Abstract

**Introduction:**

A thyroglossal duct cyst is the most common congenital anomaly of the thyroid gland and midline masses in childhood (70% abnormality in childhood, 7% in adult). Carcinomas arising from a thyroglossal duct cyst are rare (only 1% of thyroglossal duct cyst cases) and characterized by relatively non-aggressive behavior and rare lymphatic spread. They are also diagnosed mostly during the third and fourth decades of life. About 85% to 92% of all thyroglossal duct cyst carcinomas are papillary carcinomas.

**Case presentation:**

We present the case of a 44-year-old Iranian woman with Cacausian ethnicity with a painless anterior neck mass that appeared gradually over three months. She had a history of frequent painful swelling of the anterior part of her neck, which subsided with antibiotic therapy. Thyroid functional tests were normal and a thyroid scinitigraphy showed a cold nodule in the left lobe of her thyroid. A computed tomography scan revealed a large, heterogeneous enhancing soft tissue mass with cystic components in the midline of the anterior neck space. This extended from the base of the tongue,(completely separated from its muscles, to the inferior aspect of the thyroid gland and showed the destruction of the hyoid bone and the thyroid cartilage. The diagnosis of a thyroglossal duct cyst with malignant transformation was maintained. A fine needle aspiration revealed papillary carcinoma.

**Conclusion:**

This patient's case is presented because of its rare, aggressive, and invasive nature and rare and unusual manifestation, as well as its rapid increase in size, the destruction of the hyoid bone, chondrolysis of the thyroid cartilage, lymph adenopathy and the existence of a cold nodule in the thyroid gland.

## Introduction

Thyroglossal duct cysts (TDCs) are the most common anomaly in thyroid development. In general, duct cysts are benign, but 1% of cases can be malignant [1]. A review of the literature showed that 250 cases of malignant thyroglossal cysts have been reported [2]. The percentages of different types of neoplasia in reported cases of TDC are: papillary carcinoma 81.7%; mixed papillary-follicular carcinoma 6.9%; squamous cell carcinoma 5.2%; follicular and adenocarcinoma, 1.7% each; and malignant struma, epidermoid carcinoma and anaplastic carcinoma, 0.9% each [1]. Carcinomas arising from a TDC are rare and are usually characterized by non-aggressive behavior and rare lymphatic spread [3]. Most cases of TDC carcinoma are diagnosed during the third and fourth decades of life, and rarely in children under 14 years of age [4]. We present the case of a TDC papillary carcinoma because of its rarity, unusual manifestation, aggressive nature with invasion to adjacent structures, and interesting history and clinical findings.

## Case presentation

A 44-year-old Iranian woman with Cacausian ethnicity presented with an anterior midline neck mass that gradually appeared without tenderness over three months. The patient had a history of frequent painful swelling of the anterior part of her neck, which subsided with antibiotics therapy. A physical examination of the patient revealed a 100 × 55 mm mass that was painless, smooth and hard. The mass was located on the anterior part of the patient's neck and extended from the suprahyoid portion to the thyroid gland. The thyroid gland could not be separated from the mass. Thyroid functional tests (serum thyroxine, triiodothyronine and thyroid stimulating hormone) were within normal limits. A thyroid scan with technetium pertechnetate detected a cold nodule corresponding to the mass in the left lobe of the thyroid gland.

**Figure 1:**
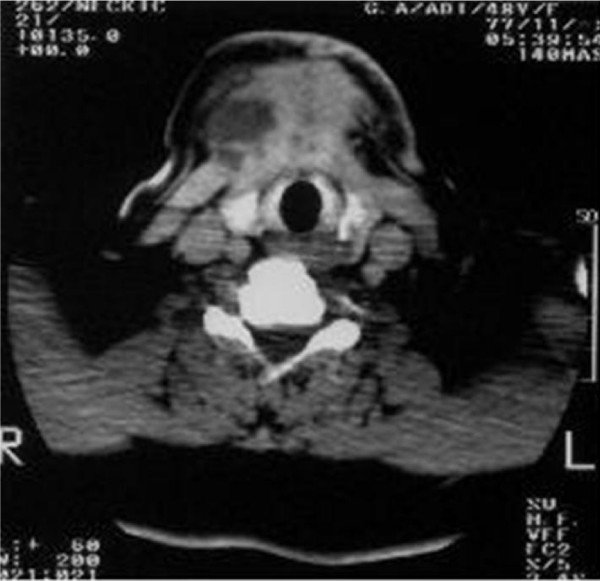
**A large heterogenous enhancing mass with cystic component in the midline portion of the anterior aspect of the neck**. Invasion of the hyoid bone is noted.

**Figure 2:**
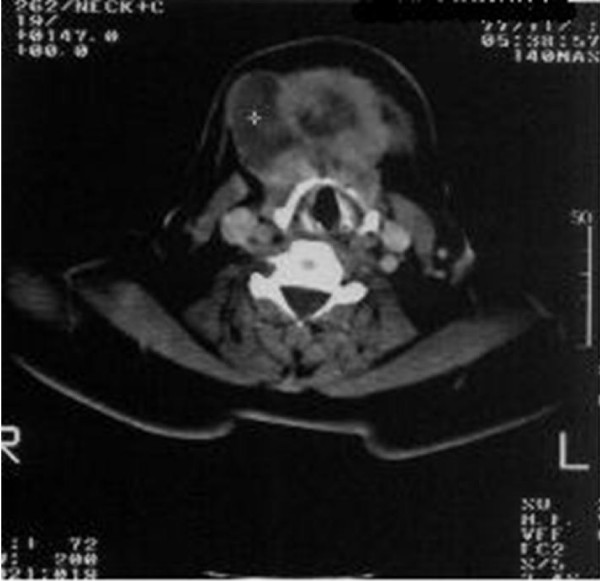
**A large heterogenous enhancing mass with cystic component in the midline portion of the anterior aspect of the neck**. Invasion of the hyoid bone is noted.

**Figure 3:**
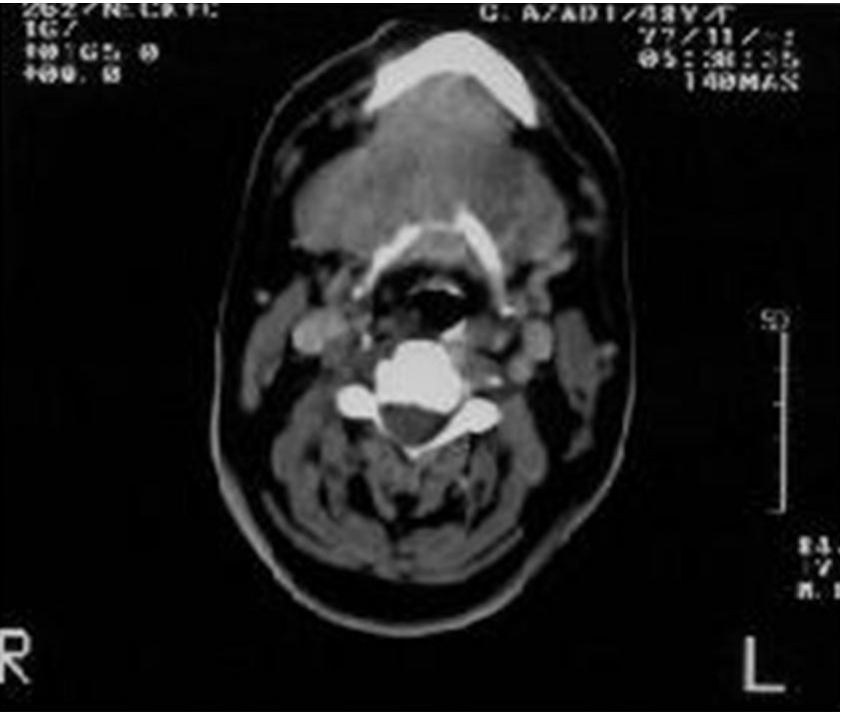
**A large heterogenous enhancing mass with cystic component in the midline portion of the anterior aspect of the neck**. Invasion of the hyoid bone is noted.

A computed tomography scan (CT) revealed a relatively large (100 × 55 × 48 mm), heterogeneous enhancing soft tissue mass with a cystic component in the midline of the anterior neck space, that extended from the base of the tongue to the inferior aspect of the thyroid gland and the bilateral aspect of the submandibular gland (Figure 1, Figure 2 and Figure 3). The mass was completely separated from the tongue muscles. The destruction of the hyoid bone and chondrolysis of the thyroid cartilage were also seen. A hypodense lesion in the left thyroid lobe and some adenopathy in the submandibular space were detected. A fine needle aspiration (FNA) revealed a papillary carcinoma. The tumor mass, together with the thyroid gland, the hyoid bone and the bilateral cervical lymph node were therefore removed. Although the thyroid gland was not involved, some micrometastases in the cervical lymph nodes were seen. The pathological report revealed a papillary carcinoma arising with a 100 × 53 mm TDC.

The pathologic report described in detail the occurrence of complex, branching and randomly oriented papillae with a central fibrovascular core and a single or stratified lining of cuboidal cells with a zoom in of 10 (×10). In addition, nuclear features showed optically clear (ground glass) nuclei and nuclear grooves (×40), indicating a malignant papillary carcinoma.

Postoperative radioactive iodine treatment and thyroid hormone supplements were recommended. The patient was followed up with a clinical examination, thyroid scintigraphy and ultrasonography of the operation site. The tumor had not recurred one year after the operation.

## Discussion

Thyroglossal duct cysts usually develop in the midline of the neck. The duct extends upward from the cyst with a few branches and secretory glands. These ducts or branches merge into a single duct at the level of the hyoid bone.

TDCs result from the dilatation of a remnant at the base of the tongue (foramen cecum), where the primitive thyroid originally descended, to its permanent location at the lower part of the neck. Failure of this tract to close predisposes the formation of a thyroglossal cyst [3-7]. Thyroglossal duct cysts most often present with a palpable asymptomatic midline neck mass at the level of or below the hyoid bone. Suprahyoid thyroglossal duct cysts are located in the midline of the neck. The more common infrahyoid thyroglossal duct cysts often have both midline and off-midline components, with the latter embedded in the strap muscles. The presence of a solid mass along it should raise the suspicion of ectopic thyroid tissue, in which occult malignancy is more likely [8].

The neck mass moves with swallowing. Some patients will have neck or throat pain, or dysphagia and the spectrum of clinical symptoms may be varied. Diagnosis is usually made clinically. Antibiotics are indicated if infection is suspected. Definitive surgical management requires excision not only of the cyst but also of the path's tract and branches [9,10].

Carcinomas arising from a TDC are rare (only 1% of reported TDC cases) and are characterized by relatively non-aggressive behavior and rare lymphatic spread [2-4]. There are different theories regarding the origin of these malignancies. Some authors believed that these carcinomas were metastases of thyroid carcinomas. However, following demonstrations of normal thyroid tissue occurrence in the wall of thyroglossal duct cysts, it is now almost universally accepted that a carcinoma may arise from thyroglossal remnants [11].

Among the vairous types of neoplasia in TDC, a papillary thyroglossal duct cyst carcinoma has the best favorable prognosis, with occurrence of metastatic lesions in fewer than 2% of cases, while a squamous cell carcinoma has the worst prognosis [12].

Regional lymph node metastasis of TDC carcinoma occur in only 7.7% of reported cases, and local invasion rarely occurs [1-4]. A rapid increase in size, the occurrence of pain, and the presence of enlarged lymph nodes may suggest malignancy [3,4]. CT scans have demonstrated unilocular or multilocular low density masses, 2 cm to 4 cm in diameter, presented anywhere from the base of tongue to the superior margin of the thyroid gland. A well-circumscribed, low density mass, and occasional peripheral rim enhancements or internal septations were seen on contrast enhanced CTs. The presence of nodules of enhancing tissue within the cyst raised the possibility of concurrent malignancy, but this may have only represented the ectopic thyroid tissue. Magnetic resonance imaging (MRI) signal characteristics vary depending on the protein contents of fluid within cysts [8].

In managing patients with carcinomas of TDC before a surgical procedure, it is important to identify whether the normally functioning thyroid tissue is in its usual location or not. Thyroid scans and thyroid function studies should be ordered preoperatively [3,8]. A CT or MRI scan is usually performed in cases of suspected thyroglossal duct cysts in adults to confirm the diagnosis and to exclude other nodal masses. Definitive surgical management requires excision not only of the cyst but also of the path's tracts and branches. The intimate association between the tract and the hyoid bone requires the simultaneous removal of the central portion of the hyoid bone to ensure the complete removal of the tract (Sistrunk procedure). Recurrence is unlikely after such an operation, except in cases with skin involvement and intraoperative cyst rupture. There is still controversy about the removal of the thyroid gland in the case of a papillary carcinoma of TDC [9-12]. A thyroidectomy is recommended in cases where: (1) the thyroid gland is found to be nodular with a cold nodule in the thyroid scan; (2) enlarged lymph nodes are present; or (3) a history of neck irradiation exists [3].

## Conclusion

This case has been presented because of the rare, aggressive and invasive nature, and rare and unusual manifestation, of the TDC described, as well as the rapid increase in its size, the associated destruction of the hyoid bone, chondrolysis of the thyroid cartilage, lymphadenopathy and the existence of a cold nodule of the thyroid gland. The patient has been disease-free one year after the operation.

## Abbreviations

CT: computed tomography; FNA: fine needle aspiration; MRI: magnetic resonance imaging; TDC: thyroglossal duct cyst;

## Consent

Written informed consent was obtained from the patient for publication of this case report and any accompanying images. A copy of the written consent is available for review by the Editor-in-Chief of this journal.

## Competing interests

The authors declare that they have no competing interests.

## Authors' contributions

LG helped in the design and coordination of the study and also contributed to writing the draft of the manuscript and interpreting the radiological figures. HM, HS and SA supervised the process of acquiring data, and also interpreted the radiological images. MA helped draft the manuscript and revised it for intellectual content. All authors read and approved the final manuscript.
